# Impact of motor self-efficacy on cyberbullying in adolescents and pre-adolescents in physical education

**DOI:** 10.3389/fpsyg.2024.1339863

**Published:** 2024-01-15

**Authors:** Jorge Rojo-Ramos, Antonio Castillo-Paredes, Noelia Mayordomo-Pinilla, Carmen Galán-Arroyo

**Affiliations:** ^1^Physical Activity for Education, Performance and Health (PAEPH) Research Group, Faculty of Sports Sciences, University of Extremadura, Cáceres, Spain; ^2^Grupo AFySE, Investigación en Actividad Física y Salud Escolar, Escuela de Pedagogía en Educación Física, Facultad de Educación, Universidad de Las Américas, Santiago, Chile; ^3^Physical and Health Literacy and Health-Related Quality of Life (PHYQoL), Faculty of Sport Science, University of Extremadura, Cáceres, Spain

**Keywords:** motor self-efficacy, ECIP-Q, E-AEM, cyberbullying, physical activity

## Abstract

**Introduction:**

In recent years, cyberbullying rates have increased, especially among adolescents in the school environment. According to the literature, the factors that influence this type of behavior are access to technologies, physical activity and BMI, among others.

**Aim:**

The aim is to find correlations between motor self-efficacy and cyberbullying.

**Methods:**

The ECIP-Q and E-AEM questionnaire was applied to 1,232 students from Spanish schools and institutes (8–18 years old) in a cross-sectional study.

**Results:**

Significant inverse correlations were found between the ECIP-Q and the E-AEM on the variables of gender, educational stage, daily physical activity, BMI, telephone ownership and hours spent on the Internet.

**Conclusion:**

In conclusion, it can be understood that the higher the level of self-efficacy, the lower the level of abuse and victimization. Therefore, physical activity could be considered to act as a regulator of cyberbullying. And it would be interesting for public administrations to increase the number of hours of physical education, to expand out-of-school physical activities and to promote an active lifestyle in order to eradicate this type of abusive school behavior.

## 1 Introduction

The lifestyle habits of citizens have changed over time, going from a more active lifestyle to a much more sedentary lifestyle. Currently, sedentary lifestyle rates in Spain are alarming; the population aged between 15 and 69 years does not meet the minimum physical activity recommendations proposed by the World Health Organization (WHO). Specifically, the latest data published by the Ministry of Health, Consumer Affairs, and Social Welfare reveal that 14% of children spend their free time on sedentary activities, which is more common in girls than in boys. In addition, 73.9% of children spent more than 1 h during the week with an electronic device, including tablets, smartphones, computers, and video games, increasing to 92.6% on weekends (Ministerio de Sanidad, 2018). Another more recent study conducted in the Spanish population revealed that 52.9% of children aged 6–10 years spend more than 2 h in front of screens for leisure and recreation purposes, rising to 67% in children aged 10–14 years, revealing an increase in screen time as they get older (Pons et al., 2021).

Unfortunately, this increased use of the Internet and social networks by children has led to the emergence of cyberbullying, a somewhat unpleasant phenomenon. This type of behavior is defined as a relatively new phenomenon that modifies traditional bullying, which is described as an unbalanced power relationship between perpetrator-victims, in which malicious actions are repeated, ranging from derogatory nicknames to degradation and manipulation of physical and material aggression (Menesini and Nocentini, 2009). Cyberbullying adapts to social networks, relying on anonymity frequently in chats or social networks, although there are other methods, such as the manipulation of photographs, which are often used in the context of cyberbullying (Roland, 2002; Hamburger et al., 2011; Gladden et al., 2014; Hinduja and Patching, 2019). This type of bullying can occur in any context (Kowalski et al., 2018), although it is much more frequent in schools and institutes, since adolescence is the period in which personality is formed and many changes occur at a physical level, establishing a sensitive period, involving the one who executes it, the one who suffers it, and the witnesses that this activity is taking place. The consequences for victims of cyberbullying are very negative, seriously deteriorating their physical and mental health because they can develop depressive disorders, anxiety, personality, eating behavior, suicidal behaviors and thoughts, self-harm, and isolation, among other types of pathologies (Moore et al., 2017; Armitage, 2021). In the European Union, studies report a cyberbullying rate ranging from 5.5 to 44%, depending on the country. In recent years, the number of cases has significantly increased. In Spain, although the rate is among the lowest in Europe, 26.65% of adolescents claim to have been victims of cyberbullying (Athanasiou et al., 2018), accounting for more than a quarter of those surveyed, and this trend has been on the rise in the last few years (Zych et al., 2016). One of the most bullied profiles at school is a student with a body mass index (BMI) above the average, with overweight and obesity being one of the groups that performs the least physical activity and has the lowest physical self-perception (Storch et al., 2006). To quantify and characterize this phenomenon, a measurement tool was developed in the form of a scale called the European Cyberbullying Intervention Project Questionnaire (ECIP-Q) (Del Rey et al., 2015). This questionnaire is composed of 22 items differentiated into two dimensions, that of the victim and that of the abuser, and is designed to identify situations of harassment or victimization.

In this sense, the scientific literature has studied the behavior of students with the aim of identifying which factors are involved in the development of this dangerous type of behavior in order to intervene effectively and improve students’ experience at school so that they can obtain the best possible learning outcomes. One of the factors studied is perceived self-efficacy, defined as the perception and feeling of capability regarding the tasks and challenges that appear in people’s daily lives and their ability to face and overcome them satisfactorily, controlling these situations (Hernández-Álvarez et al., 2011). With the aim of developing a tool that allows the measurement of this construct and enables comparison with other variables, the (E-AEM), a scale composed of 10 items, was developed (Hernández-Álvarez et al., 2011). Self-efficacy has been studied as a determining factor in academic performance, finding positive correlations in fields such as mathematics and language (Hayat et al., 2020; Luo et al., 2023), in the field of physical activity, specifically in motor self-efficacy, it has been determined that the perception of self-efficacy acts as a mediator in the regulation of behavior, directly associated with the practice of physical activity in youth and, therefore, with the appearance of aggressive or undesired behaviors (Welk and Schaben, 2004; Greco, 2021). Authors who have studied the possible relationships between physical activity and behavior have found that physical activity is an ideal and effective means for the transmission of prosocial and positive values, while those who practice physical activity on a recurrent basis are less likely to develop problematic behaviors, including a lower probability of bullying and cyberbullying (Portolés Ariño and González Fernández, 2015; González et al., 2016; Arcila-Arango et al., 2022). In addition, negative correlations have been found between the practice of physical activity and bullying related to the physical appearance and weight of victims, where those with a higher BMI practice less physical activity (Storch et al., 2006; Losekam et al., 2010), establishing a high correlation between BMI and the likelihood of teasing by peers (Ievers-Landis et al., 2019).

The life stage in which this type of bullying occurs most often is childhood and adolescence. Studies have examined the factors that predict cyberbullying, finding a correlation with age, although only in adolescence, since older adolescents tend to perpetrate it more than younger adolescents and children, although in adulthood it is only higher in younger adults (Yudes et al., 2020). Since cyberbullying has become a highly prevalent social problem that greatly affects the mental health of adolescents, it is important to identify the factors involved in the development and profile of those who suffer it, with the intention of developing strategies to help reduce this incidence and the effects of this harmful practice. Therefore, this study aimed to explore the possible correlations between motor self-efficacy and cyberbullying, using the E-AEM scale and the ECIP-Q questionnaire, as a function of different variables: gender, educational stage, BMI, and the use and possession of electronic devices.

## 2 Materials and methods

### 2.1 Design

This work was designed as a cross-sectional study as defined in Setia (2016). It was carried out in schools and institutes in the autonomous community of Extremadura, Spain, during the year 2022.

### 2.2 Participants

According to the Census Report provided by the National Institute of Statistics,^1^ there are 43,043 inhabitants between 8 and 18 years of age in the autonomous community of Extremadura (Spain). The sample size was 1,232 participants exceeding 381 participants for a Confidence Level of 95%, with a margin of error of ±5%. A non-probability convenience sampling technique was used to select the appropriate sample size (Salkind, 1999). The final sample consisted of 1,232 students from schools and institutes of the community of Extremadura, belonging to primary education (from 8 to 11 years old), secondary education (from 12 to 16 years old), or high school (from 16 to 18 years old). Of the total number of students, 49.1% were boys and 50.9% were girls, establishing a fairly balanced sample with respect to gender. Most of the participants stated that they had a smartphone (Yes = 97.9%; No = 2.1%) and that they had access to different electronic devices with Internet connections (Yes = 90.7%; No = 1.5%; Sometimes = 7.7%). The characteristics of the sample are listed in Table 1.

**TABLE 1:** Sociodemographic profile of physical education students (*N* = 1232).

Variables	Categories	*N*	%
Gender	Boy	605	49.1
	Girl	627	50.9
Age	8–11 years	64	5.2
	12–15 years	752	61.0
	16–18 years	416	33.8
Do you have a smartphone?	Yes	1206	97.9
	No	26	2.1
Do you have a computer, tablet or other device with internet connection?	Yes	1118	90.7
	No	19	1.5
	Sometimes	95	7.7
Do you think there is cyberbullying in your school?	Yes	261	21.2
	No	270	21.9
	I don’t know	701	56.9
Have you ever suffered a situation of cyberbullying by a classmate?	Yes	118	9.6
	No	1114	90.4
Do you think that people who suffer cyberbullying have a negative influence on their academic performance?	Yes	1097	89.1
	No	22	1.8
	I don’t know	113	9.2

N: number, %: percentage.

### 2.3 Instruments

**Sociodemographic Questionnaire:** To collect sociodemographic data from the sample, they were provided with a specific questionnaire containing questions on the variables shown in Table 1: age, sex, possession of a smartphone, access to electronic devices with Internet connections, such as computers and tablets, cyberbullying, and physical activity.

**The European Cyberbullying Intervention Project Questionnaire (ECIP-Q)**: This scale, validated by Del Rey et al. (2015), is composed of 22 items divided into two dimensions: (1) that of the bully, composed of 11 items aimed at measuring the frequency, duration, and severity of cyberbullying actions (example: 1- I have said swear words to someone or insulted them using SMS or Internet messages; and (2) the victim, composed of 11 items aimed at measuring the emotional and behavioral responses that appear as a consequence of cyberbullying (example: 1- Someone has said swear words to me or insulted me using email or SMS; and (2) Someone has hacked my account and impersonated me). For quantification, the questionnaire used a Likert-type scale ranging from 0 (never) to 4 (always). The authors (Del Rey et al., 2015) reported a reliability value of Cronbach’s alpha coefficient: total = 0.87, victimization = 0.80, aggression = 0.88).

**Motor Self-Efficacy Scale (E-AEM):** This instrument was validated in Spanish for school-aged children by Hernández-Álvarez et al. (2011) consists of 10 items that present possible situations experienced during physical sports practice (e.g., Item 1: During a sports game, I can get into trouble even if someone opposes me; Item 7: Whatever happens during a sports game, I am usually able to handle the situation). Respondents indicated their level of agreement on a Likert-type scale, with values ranging from 1 (“strongly disagree”) to 4 (“strongly agree”). Scores were calculated by summing up the 10 items, with possible scores ranging from 10 (indicating a low level of motor self-efficacy) to 40. The instrument has a reliability value of 0.82 based on Cronbach’s alpha coefficient.

### 2.4 Procedure

In order to collect a sample for the study, the researchers accessed the database of the Department of Education and Employment of the Regional Government of Extremadura to identify schools where physical education (PE) is taught to students aged 8–18. They then contacted PE teachers at these schools by email, explaining the study’s objectives and requesting that they arrange for a researcher to visit the school to administer a questionnaire on cyberbullying to students whose parents had provided informed consent. The questionnaire included sociodemographic questions as well as the ECIP-Q and E-AEM scales. Students were given a tablet to access the questionnaire via a Google Form, and each question was explained to them to avoid any confusion. Once all the questionnaires were collected, the researchers processed and anonymized the data before passing it on to another researcher for blind analysis.

In order to carry out this research, a protocol was adhered to following the considerations of the Declaration of Helsinki and this protocol was approved by the Biosafety and Bioethics Committee of the University of Extremadura in Spain (Registration Code 72/2022).

### 2.5 Statistical analysis

SPSS statistical software version 23 for MAC (IBM SPSS, Chicago, IL, USA) was used to process the collected data. First, the Kolmogorov-Smirnov test was used to explore the assumption of normality in the distribution of continuous variable data. It was found that this assumption was not met, so nonparametric statistical tests were used. Spearman’s Rho test was used to analyze the relationship between each of the ECIPQ dimensions and the E-AEM scores. To interpret the correlation coefficients, the thresholds proposed by Mondragón Barrera (2014), were followed: rom 0.01 to 0.10 (low correlation), from 0.11 to 0.50 (medium correlation), from 0.51 to 0.75 (considerable correlation), from 0.76 to 0.90 (very high correlation) and from 0.91 to 1.00 (perfect correlation). Cronbach’s alpha and McDonalds omega were used to analyze the reliability of each instrument. To interpret the values of the reliability test, we took as a reference those set out by Nunnally and Bernstein (1994): <0.70 (low), 0.71–0.90 (satisfactory) and >0.91 (excellent). To ensure the integrity and quality of the data, to give robustness to the accuracy of trends and patterns within our study sample, an outlier study was conducted that involved detailed inspection of extreme values for all items in individual responses. Those responses whose values in all items of the scale (32) were extreme were eliminated.

## 3 Results

Spearman’s Rho test was used to analyze the relationship between the different dimensions of the ECIPQ and the E-AEM scores (Table 2). Significant inverse associations were found between the two dimensions of ECIPQ and E-AEM scores. However, the ECIPQ dimension concerning the victim showed a low correlation, whereas the second dimension concerning the abuser exhibited a moderate correlation. According to gender, all correlations were inverse, showing higher correlations in male students. In contrast, female students did not show significant associations between the first dimension of the ECIPQ and the E-AEM. Similarly, inverse correlations were found when evaluating the associations according to the educational stage to which the student belonged, manifesting higher correlations in the primary education stage; however, the only non-significant result was manifested when correlating the first dimension of the ECIPQ and E-AEM in the primary education stage.

**TABLE 2:** Correlation between ECIPQ dimensions and E-AEM scores according to student gender and educational stage.

Dimensions	E-AEM *ρ (p)*	Gender		Educational stage	
		**Men**	**Women**	**Primary**	**Secondary**
(1) ECIPQ-Victim	−0.08 (<0.01)**	−0.12 (<0.01)**	−0.03 (0.53)	−0.19 (0.11)	−0.07 (0.02)*
(2) ECIPQ-Abuser	−0.11 (<0.01)**	−0.13 (<0.01)**	−0.09 (0.03)*	−0.27 (0.02)*	−0.10 (<0.01)**

The correlation is significant at the **p < 0.01; *p < 0.05. Each score obtained is based on a Likert scale: E-AEM (1–4): 1 (strongly disagree) to 4 (strongly agree). ECIP-Q (0–4): 0 (never) to 4 (always).

Regarding the relationship between the ECIPQ and E-AEM (Table 3), the daily PA performed by the students generated disparate results. On the one hand, students who performed less than 60 min did not show significant correlations between the ECIPQ and E-AEM. However, those who performed more than 60 min daily showed inverse and medium correlations, although the only significant association was between the second dimension and E-AEM score. Similarly, underweight students showed significant mean and inverse correlations between both ECIPQ and E-AEM dimensions. Similarly, students with a healthy weight showed low inverse correlations, significant only in the second dimension of the ECIPQ. Finally, in overweight children, the trend was reversed with positive correlations; however, none of these correlations were significant.

**TABLE 3:** Correlation between the ECIPQ dimensions and the E-AEM score as a function of hours of daily PA and BMI.

Dimensions	E-AEM *ρ (p)*	Hours of daily physical activity		BMI		
		**<60 min**	**>60 min**	**<18.5**	**18.5–24.9**	**≥25**
ECIPQ-Victim	−0.08 (<0.01)**	0.03 (0.75)	−0.34 (0.37)	−0.18 (<0.01)**	−0.07 (0.06)	0.16 (0.07)
ECIPQ-Abuser	0.11 (<0.01)**	−0.14 (0.14)	−0.11 (<0.01)**	−0.17 (<0.01)**	−0.10 (<0.01)**	0.01 (0.93)

The correlation is significant at the **p < 0.01. Each score obtained is based on a Likert scale: E-AEM (1–4): 1 (strongly disagree) to 4 (strongly agree). ECIP-Q (0–4): 0 (never) to 4 (always).

Table 4 shows the correlations between both scales when considering the daily hours spent in front of technological devices and students’ possession of a cell phone. Students who spent less than an hour and a half in front of a device showed significant and average inverse correlations. By contrast, those who exceeded the general recommendations showed a low and significant inverse association. Likewise, students who do not have a cell phone show inverse, medium and significant associations, while those who do have a mobile phone also show inverse and significant, but low associations.

**TABLE 4:** Correlation between ECIPQ dimensions and E-AEM scores as a function of daily hours in front of a technological device and owning a telephone.

Dimensions	E-AEM *ρ (p)*	Hours per day in front of computer/tablet/mobile phone		Own telephone?	
		**<90 min**	**>90 min**	**No**	**Yes**
ECIPQ-Victim	−0.08 (<0.01)**	−0.22 (0.20)*	−0.06 (0.04)*	−0.47 (0.02)*	−0.07 (0.02)*
ECIPQ-Abuser	−0.11 (<0.01)**	−0.32 (<0.01)**	−0.09 (<0.01)**	−0.43 (0.03)*	−0.10 (<0.01)**

The correlation is significant at the **p < 0.01; *p < 0.05. Each score obtained is based on a Likert scale: E-AEM (1–4): 1 (strongly disagree) to 4 (strongly agree). ECIP-Q (0–4): 0 (never) to 4 (always).

Finally, the reliability values of Cronbach’s alpha and McDonalds’ omega for each of the dimensions studied are presented in Table 5. Thus, the values of the ECIPQ were considered satisfactory and those of the E-AEM, excellent.

**TABLE 5:** Reliability values of Cronbach’s alpha and McDonalds’ omega.

Dimensions	Cronbach’s alpha	McDonalds’ omega
ECIPQ-Victim	0.874	0.865
ECIPQ-Abuser	0.877	0.861
E-AEM	0.907	0.909

## 4 Discussion

The aim of this study was to search for and identify correlations between the ECIP-Q questionnaire and the E-AEM scale, as well as to compare these scores between gender, educational stage, BMI, hours of daily physical activity, time dedicated to technologies, and possession of these technological devices.

First, an inverse and significant correlation was found between the E-AEM scale scores and both dimensions of the ECIP-Q questionnaire of average character in the abuser, which means that the higher the score on the motor self-efficacy scale, the lower the score on this dimension, establishing that the abuser is less likely to commit actions of this style if he/she has higher motor self-efficacy. In this sense, authors who have explored the behaviors of students in relation to motor self-efficacy have found results in line with what has been described, establishing that a better motor self-efficacy could be related to a better regulation of behavior and an increase in empathy towards peers, decreasing this type of disruptive behavior (Greco, 2021; Arcila-Arango et al., 2022). Other studies have found that a higher level of self-efficacy can reduce the likelihood of students being victimized by cyberbullying. This can be considered a useful tool for preventing disruptive behavior (Kokkinos et al., 2015). According to these results, designing strategies that increase students’ motor self-efficacy would reduce cyberbullying rates because there would be fewer abusive behaviors and fewer victims of this phenomenon, thus improving students’ coexistence. Regarding the gender variable, significant inverse correlations were found in both genders and dimensions, except in the first dimension of the ECIP-Q in females. These associations were higher in males, with an average character in both dimensions. These results coincide with those obtained in other studies conducted in the exploration of motor self-efficacy, where boys had higher self-efficacy than girls (Hernández-Álvarez et al., 2011; Chen et al., 2019; Ortiz Gómez, 2021). In the same way, the correlation is higher in boys than in girls in both dimensions; this behavior can be explained by what is found in the scientific literature, which suggests that boys are more likely to occupy the role of abusers than girls (Smith et al., 2019), although there are discrepancies about which gender tends to occupy the role of the victim more (Chocarro de Luis and Garaigordobil Landazabal, 2019; Smith et al., 2019). Regarding educational stage, the results show a significant inverse association in both dimensions in the secondary stage and in the second dimension in the primary stage. Students in primary education obtain a higher correlation in general, with motor self-efficacy gaining more importance; these results express that self-efficacy decreases with age, and the significant medium inverse correlation of the abuser dimension of the ECIP-Q explains that, the higher the self-efficacy, the lower the incidence of this role in this educational stage. The authors agree that the decrease in self-efficacy with age may be due to the self-esteem problems reported in adolescence (Hernández et al., 2008; Velázquez Buendía et al., 2015; Perea Chafé et al., 2016). This is a sensitive period in which personality and social circles are formed, making this population prone to a decline in self-esteem and the appearance of these risky behaviors. However, motor self-efficacy can act as a regulator of this behavior.

Continuing the results of the exploration between the dimensions of the ECIP-Q and E-AEM scores as a function of physical activity and BMI, significant correlations were found only in students who performed >60 min of physical activity per day, with an inverse and average character. Therefore, those who performed more than 60 min of physical activity per day had greater motor self-efficacy and scored lower on the ECIP-Q abuser dimension. The results obtained by the scientific community support those obtained in this work, stating that physical activity, in addition to being an emotional regulator and ideal means for the promotion of values, is an effective tool for implementing programs that decrease the incidence of cyberbullying (García-Hermoso et al., 2020; Benítez-Sillero et al., 2021; Benitez-Sillero et al., 2022). Additionally, other studies have determined that students who are more physically active have less time to use phones or other technologies, so these rates of cyberbullying would also decrease Relative to BMI, the results show significant correlations of inverse nature and mean magnitude in both dimensions of the ECIP-Q in students with a BMI below 18.5 and those with a BMI between 18.5 and 24.9 in the bully dimension. This correlation implies that students who are in the underweight range and have higher scores on motor self-efficacy are less likely to be in the role of bullies or victims of cyberbullying, and those in the normo weight on the bully dimension. Studies on the relationships between BMI, motor self-efficacy, and cyberbullying are scarce, and studies carried out to date have examined the influence of overweight and obesity in both roles, determining that both increase the probability of being a victim of cyberbullying (Lee et al., 2018; Carvalho et al., 2021), although the results of this study did not show significant associations in people with a BMI over 25.

Finally, regarding the correlations between the dimensions of the ECIP-Q and E-AEM according to the time spent on technologies and the possession of a smartphone, significant correlations were obtained in all dimensions and variables. The strongest associations occur in those who spend less than 90 days with electronic devices and do not have their own phones, both in the abuser and victim dimensions. Research published in this area shows similar results, identifying as risk factors the time spent on screens and the possession of one’s own phone and Internet, establishing that the more time, the greater the probability of suffering and executing it (Chen et al., 2017; You and Lim, 2018; Redondo Pacheco, 2023).

Lastly, the results showed that the higher the motor self-efficacy, the lower the perpetration of cyberbullying, both in the dimension of abusers and victims in different populations. Knowing how the variables behave helps to understand the profiles and behaviors of this phenomenon, establishing the lines of action that should be taken in the future to curb these situations. In this sense, studies on motor self-efficacy report that it is higher in those who are physically active, as in the results obtained in this paper, establishing this variable as a useful tool to reduce cyberbullying situations through prosocial values and the use of more time exercising, being farther away from social networks and the Internet (García Puello et al., 2020; Sheikh et al., 2022).

### 4.1 Practical applications

The results of this study reveal information about the research gap, knowing the correlations and behaviors of the variables that influence the appearance of these behaviors, contributing to the knowledge of the factors involved in cyberbullying, both in the role of abusers and victims, with the aim of promoting practices that prevent the occurrence of these behaviors. The results obtained in this study show that those who engage in more physical activity have more motor self-efficacy and are therefore less likely to perpetrate the behaviors that characterize cyberbullying as well as being less likely to suffer it. Physical activity is also related to healthier lifestyles, with a lower incidence of overweight and obesity, which is one of the most influential factors in bullying. Thus, it seems logical to propose that the promotion of physical activity is an effective means of reducing cyberbullying rates. This promotion could be done from educational institutions through physical education or from other institutions, such as municipalities, with physical activity workshops that promote positive values and awareness of this type of behavior.

### 4.2 Limitations and future lines

The results obtained in this study should be interpreted with caution, as they present some limitations. Since this is a cross-sectional study, causal relationships cannot be established, and electronic questionnaires were used, which have both advantages and disadvantages. Moreover, a non-random sampling method based on convenience sampling was used; therefore, the results should be interpreted with caution. Finally, caution must be taken in the interpretation of the results, since the sample belongs only to the community of Extremadura, and there may be cultural factors that influence the results obtained. In the future, it would be interesting to replicate the study in other communities to increase the sample to other contexts; also, it would be interesting to repeat this study in other samples to obtain data on the variables of motor self-efficacy and cyberbullying, since the scientific literature on this topic is quite scarce, and it is difficult to compare the results.

## 5 Conclusion

The ECIP-Q and E-AEM scales were used to measure motor self-efficacy and cyberbullying bully-victim behavior. The results showed an inverse correlation (for all variables), whereby the higher the motor self-efficacy, the lower the incidence of both dimensions of cyberbullying, measured in different populations. In general, boys scored higher on the E-AEM scale and had a higher correlation with cyberbullying, as did primary school boys than secondary school boys. Moreover, those who performed more than 60 min of daily physical activity had a higher score in motor self-efficacy, although only in the bullying dimension. With respect to BMI, they are the ones who have underweight a higher correlation in both dimensions, without finding significant correlations in those with a BMI greater than 25. Finally, the strongest associations of an inverse nature are higher in those who do not have their own telephone and spend less time on technologies. Based on these results, it is worth considering that it is necessary to implement strategies to ensure that they comply with the maximum recommended time in front of screens, one of which is physical activity, as it increases motor self-efficacy and keeps them away from screens for the duration of the activity. On the other hand, it is a means to promote positive values that could increase empathy and decrease the incidence of cyberbullying.

## Data availability statement

The raw data supporting the conclusions of this article will be made available by the authors, without undue reservation.

## Ethics statement

The studies involving humans were approved by the Bioethic Comitée of University of Extremadura (Spain). The studies were conducted in accordance with the local legislation and institutional requirements. Written informed consent for participation in this study was provided by the participants’ legal guardians/next of kin. The use of the data in this study did not require the approval of an accredited ethics committee as it is not covered by data protection principles, i.e., it is non-identifiable and anonymized data collected through an anonymous survey for elite athletes. Furthermore, under Regulation (EU) 2016/679 of the European Parliament and of the Council of 27 April 2016 on the protection of individuals with regard to the processing of personal data and on the free movement of such data (which entered into force on 25 May 2016 and is binding as of 25 May 2018), data protection principles need not be applied to anonymous information (i.e., information relating to an identifiable natural person, nor to data of a subject who is not, or is no longer, identifiable). Therefore, the Regulation does not affect the processing of our information. Even for statistical or research purposes, its use does not require the approval of an accredited Ethics Committee.

## Author contributions

JR-R: Data curation, Investigation, Methodology, Project administration, Supervision, Writing – original draft, Writing – review and editing. AC-P: Funding acquisition, Investigation, Methodology, Resources, Visualization, Writing – original draft, Writing – review and editing. NM-P: Conceptualization, Formal Analysis, Investigation, Writing – original draft, Writing – review and editing. CG-A: Investigation, Project administration, Supervision, Visualization, Writing – original draft, Writing – review and editing.

## Footnotes

1
www.ine.es
